# Bioderived Ionic Liquids and Salts with Various Cyano Anions as Precursors for Doped Carbon Materials

**DOI:** 10.3390/ijms221910426

**Published:** 2021-09-27

**Authors:** Alina Brzęczek-Szafran, Bartłomiej Gaida, Agata Blacha-Grzechnik, Karolina Matuszek, Anna Chrobok

**Affiliations:** 1 Faculty of Chemistry, Silesian University of Technology, Krzywoustego 4, 44-100 Gliwice, Poland; bartlomiej.gaida@polsl.pl (B.G.); Agata.Blacha-Grzechnik@polsl.pl (A.B.-G.); anna.chrobok@polsl.pl (A.C.); 2 School of Chemistry, Monash University, Clayton, VIC 3800, Australia; karolina.matuszek@monash.edu

**Keywords:** carbohydrate, sugar, ionic liquid, biomass, bio-ILs, N-doped carbon, mesoporous carbon, oxygen reduction reaction

## Abstract

Carbohydrate moieties were combined with various cross-linkable anions (thiocyanate [SCN], tetracyanoborate [TCB], tricyanomethanide [TCM], and dicyanamide [DCA]) and investigated as precursors for the synthesis of nitrogen-doped and nitrogen-/sulfur-co-doped carbons. The influence of the molecular structures of the precursors on their thermophysical properties and the properties of the derived carbon materials was elucidated and compared to petroleum-derived analogs. A carbohydrate-based ionic liquid featuring an [SCN] anion yielded more carbon residues upon carbonization than its 1-ethyl-3-methylimidazolium analog, and the resulting dual-doping of the derived carbon material translated to enhanced catalytic activity in the oxygen reduction reaction.

## 1. Introduction

Due to the current focus on the development of more sustainable and ecofriendly processes, biomass has been intensively exploited to prepare high-value-added chemicals and materials. In addition to the continuous search for useful biomass feedstock and ecofriendly processes suitable for its derivatization, products such as bio-derived solvents, additives, and functional materials are also of special interest. In this regard, bio-derived ionic liquids (ILs) [1,2,3] that can serve as solvents for the biomass dissolution desirable in the closed-loop biorefinery concept [4], as solvents for ionothermal biomass upgrading into functional carbon materials, or as carbon precursors themselves [5,6,7] can be useful sustainable chemicals.

The doping of carbon materials with electron-donating or electron-withdrawing heteroatoms (N, B, P, F, or S) provides a convenient way to tune and control their physicochemical properties, i.e., by increasing their electrical conductivity, basicity, oxidation stability, and catalytic activity (both electrochemical and chemical). Doped carbon materials have recently gained a great deal of interest as metal-free electrode materials for electrochemical applications [8] (electrocatalysis, Li-ion batteries, or supercapacitors) and as metal-free catalysts for traditional chemical processes (aerobic oxidation of alcohols [9], acetylene hydrochlorination [10], or the Knoevenagel condensation [11]).

There is particular interest in the carbonization of biomass, which can serve as an inexpensive, abundant, and renewable carbon source. Various feedstocks, including saccharides, cellulose, and lignin are used to yield carbon residues from biological, chemical, or thermochemical processes [12]. One of the strategies to provide biomass-derived carbon materials with specific properties, such as enhanced surface area or heteroatom doping, is the use of ILs, which can act as heteroatom sources, stabilizers, pore-generating agents, or as precursors themselves, in the carbonization process [5,13,14].

Bio-derived ILs have recently been studied as alternatives to conventional ILs derived from fossil fuels, ready to develop sustainable doped carbon materials. They combine the properties of biomass, such as high carbon content, with the properties of ILs, including high thermal stability, low vapor pressure, and nitrogen-rich molecular structures. Under thermal treatment, N atoms can be uniformly introduced into the carbon structure during the in situ self-assembly process [6]. Various feedstocks, including amino acids [7,15,16], carbohydrates [5], glucamine [6], alginic acid [17], cellulose [18], and furfurylamine [19], have been transformed into ILs and further utilized as N-doped carbon precursors. Via precise design of the structure of the precursors at the molecular level, the final properties of the carbonaceous material, such as the N-doping level, carbonization yield, and the surface properties, can be controlled [5,20]. So far, protic ILs have been shown to be the most viable for the carbonization process due to their easy and inexpensive synthesis [21]. However, carbon materials with the highest N-doping were synthesized from ILs containing nitrile groups, which, under thermal treatment, form polytriazine networks [20]. It has been demonstrated that the ionic structure, together with the presence of the N-based cation- and anion-containing cyano groups, contributes to the formation of carbon residues [22]. When nonionic reactants were subjected to thermolysis, they yielded only volatile products with no carbon residues, even though they possessed cross-linkable moieties in their structure [23]. ILs with both cross-linkable cations and anions were investigated as precursors for nitrogen-rich nanoporous carbons [23,24,25,26,27]. Conventional imidazolium-based ILs combined with tetracyanoborate anions yielded carbon with a nitrogen content of up to 17.5% at 1000 °C [26]. With the interplay of the anion, carbon residues with nitrogen doping of up to 26% were synthesized at 800 °C using the dicyanamide anion. Moreover, not only did the cross-linkable anion influence the properties of the final carbon materials, but so did the cation, as shown by replacing the *N*,*N*-ethylmethyl-imidazolium cation with 3-methyl-*N*-butyl-pyridinium (at the same carbonization temperature, material with a 10% lower nitrogen content was synthesized) [23].

**Figure 1:**
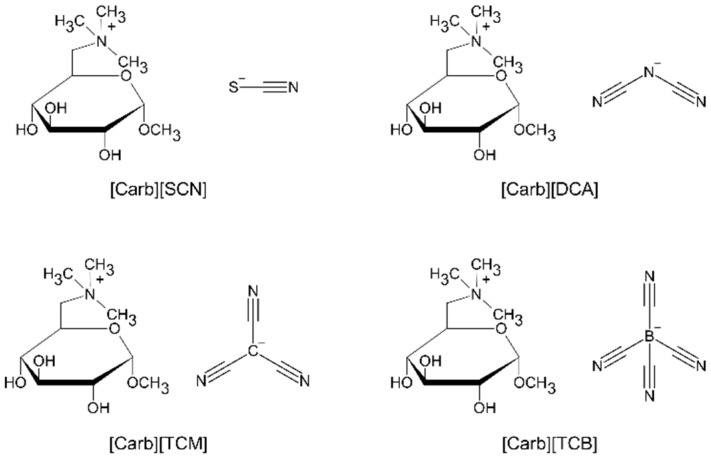
Molecular structures of the investigated carbohydrate-derived ionic liquids and salts with various cyano anions.

In this study, we combined carbohydrates with different cross-linkable anions (Figure 1) to obtain quaternary ammonium compounds [28], and we investigated their potential as nitrogen-doped carbon precursors, being an alternative to traditional imidazolium-based ILs. To test the influence of the precursor structure on the properties of the carbon materials, such as carbon yield and heteroatom doping, we synthesized a series of ionic precursors composed of a glucose moiety-based cation and four cyano-based anions. The resulting carbon materials were utilized as electrocatalysts for the oxygen reduction reaction process. The limits of the adopted approach were elaborated on and discussed.

## 2. Results and Discussion

### 2.1. Synthesis of the Precursors and Their Thermophysical Properties

**Figure 2:**
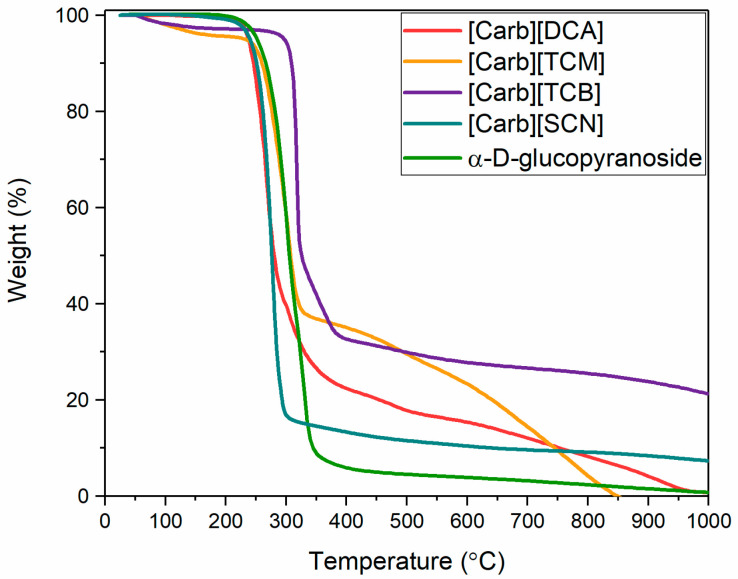
Thermogravimetric analysis curves of carbohydrate-based ILs and salts with cyano anions.

A series of carbohydrate-derived ILs and salts with various cyano anions (Figure 1) was synthesized via the bromination of commercially available methyl α-d-glucopyranoside with tetrabromomethane via the Appel reaction, followed by quaternization and anion exchange, according to a previously described procedure [5]. For the anion exchange, silver salts of tetracyanoborate (TCB), tricyanomethanide (TCM), dicyanamide (DCA), and thiocyanate (SCN) were used. The final products were ILs ([Carb][TCB], [Carb][TCM]) with melting temperatures <100 °C or salts ([Carb][DCA], [Carb][SCN]) with melting temperatures >100 °C. The thermal properties of the products, which were investigated by differential scanning calorimetry (DSC) and thermogravimetric analysis (TGA), are compiled in Table 1 and Figure 2.

The new ionic systems showed carbohydrate-like, supercooled thermal behavior; once melted, they did not undergo a reversible crystallization, but showed a glass transition, as shown in repetitive heating and cooling experiments conducted with DSC (Figure S3). The combination of α-d-glucopyranoside-based cation with cross-linkable cyano-based anions, such as [SCN], [DCA], and [TCM], had very little influence on the thermal stability of the systems, which gave *T*_onset_ at 229 °C, 232 °C, and 255 °C respectively. In contrast, the [TCB] anion improved the thermal stability of the parent carbohydrate by 65 °C (*T*_onset_ = 308 °C). Therefore, the IL with the [TCB] anion had the highest stability when compared to the carbohydrate-derived ILs reported in the literature, except for the bis(trifluoromethanesulfonyl)imide [NTf_2_] [29,30] and trifluoromethanesulfonate [OTf] [31] anions. On the other hand, comparing the thermal properties of the investigated carbohydrate-based ILs to their 1-ethyl-3-methylimidazolium analogs ([C_2_mim][DCA], [C_2_mim][SCN], [C_2_mim][TCM]) [32] and 1-*n*-butyl-2,3-dimethylimidazolium analog ([C_4_mmi][TCB]) [33], the latter had higher thermal stability, which is a general trend for imidazolium-based ILs with various anions [1].

### 2.2. Carbonization of Carbohydrate-Derived ILs and Salts

**Figure 3:**
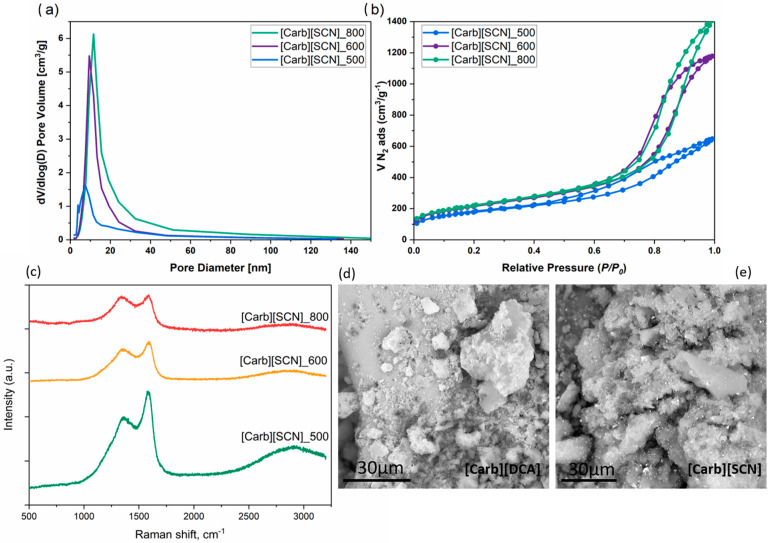
(**a**) Pore-size distribution and (**b**) N_2_ gas adsorption/desorption isotherms for carbons derived from the precursor [Carb][SCN] at 500, 600, and 800 °C. (**c**) Raman spectra. (**d**,**e**) SEM images of carbon materials: [Carb][SCN], [Carb][DCA].

The α-d-glucopyranosides combined with different cross-linkable anions were further used as precursors for doped carbon materials. As reported previously, cyano-based anions such as [DCA], [TCM], and [TCB] contribute very little to pore generation in the carbonization process [5,20], unlike bulky anions such as [NTf_2_] [34]; hence, the precursors were mixed with silica nanoparticles, serving as a hard template, prior to the carbonization step. The obtained gels were carbonized at 500, 600, or 800 °C in a tube furnace with a heating time of 2 h. The template was removed after the carbonization step, affording mesoporous materials (Figure 3d,e) with specific surface areas of S_BET_ ~700 m^2^·g^−1^, confirmed by their N_2_ adsorption–desorption isotherms and possessing an average pore size of 8–11 nm, as shown for materials derived from precursor with [SCN] anions (Figure 3a,b). The Raman spectra of the materials prepared at various temperatures (500–800 °C) from the [Carb][SCN] precursor (Figure 3c) exhibited a characteristic disorder-induced band (D-band at ~1350 cm^−1^), ascribed to structural defects, and a graphite band (G-band at ~1590 cm^−1^), corresponding to an optical phonon mode with *E_2g_* symmetry associated with an in-plane stretching of the *sp^2^*-bonded carbon atoms [35]. With the increase in the carbonization temperature, an increase in the D intensity (*I_D_*) with respect to the G intensity (*I_G_*) was observed. The intensity ratio (*I_D_*/*I_G_*) was 0.78, 0.84, and 0.96 for the materials carbonized at 500, 600, and 800 °C, respectively. This counterintuitive characteristic of the carbonization process, usually associated with graphitization, was observed previously for various carbohydrates (saccharose and cellulose) [36]. The increase in the D band is related to the disorder of the char structure which consists of very small polyaromatic units, yielded under the release of heteroatoms [36].

Both the carbonization temperature and the precursor structure affected the elemental composition of the produced samples (Table 2). Materials with a maximum nitrogen doping of up to 11.1% were synthesized at 600 °C (from the precursors containing [DCA] and [TCM] anions), and doping up to 8.7% was noted for a material synthesized at 800 °C (from the precursor containing the [DCA] anion). The combination of the carbohydrate moiety with the [SCN] anion yielded materials with additional sulfur doping, ranging from 4.7% to 2.6%, depending on the carbonization temperature.

Comparing the doping levels achieved for materials derived from sugar-based salts with their pyridinium or imidazolium analogs, the latter yielded carbon residues with higher N-doping (carbon residues with a nitrogen content as high as 23–24% were achieved from [C_2_mim][DCA] and [C_2_mim][TCB] precursors at 800 °C) [26,27]. However, it is impossible to compare the doping levels accurately due to the use of the silica template in the annealing process of the sugar-based precursors. The hard template alters the reaction mechanisms, resulting in lower doping values than in the bulk material; for instance, the N-doping of material derived from [Carb][DCA] was 9.6% [5],whereas, when it was carbonized in the presence of a template, the value was only 8.7%. For thiazolium salts, even higher discrepancies were reported, with up to 3% and 5% for nitrogen and sulfur, respectively [24]. Nevertheless, the lower doping levels observed for carbohydrate-derived precursors may be ascribed to the nonaromatic cation facilitating precursor decomposition and a lower N/C ratio in the molecular structure [23,26].

Investigating the carbonization yields, besides the high carbon content in the carbohydrate-derived precursors, only the precursor possessing the [SCN] anion yielded more carbon residues than its imidazolium analog [C_2_mim][SCN] [27]. Nevertheless, the thiocyanate anion yielded the least carbon out of all the investigated anions due to its trimerization into thiocyanuric acid, which does not form extended polymer networks and undergoes sublimation instead [27]. The final carbon yields resulted mostly from the cross-linkable anions (Table 2). Comparing the carbonization yield of sugar-based cations combined with [DCA], [TCM], and [TCB] anions with traditional imidazolium analogs, the latter yielded more carbon residues, which can be ascribed to their aromatic cation structure. Even though the cations without cross-linkable functional groups mostly decompose during thermal treatments [20], the aromatic moieties in the structures of the precursors contribute to higher carbon yields, by being confined in the three-dimensional carbonaceous frameworks formed during the cyclotrimerization reaction of the anions [20].

Comparing the mass loss directly from the thermogravimetric analysis, [C_2_mim][SCN] already yielded only 2.5% of ash at 450 °C, and no further decrease in yield was observed up to 850 °C [32], while [Carb][SCN] yielded 12% of carbon residues at 450 °C and 8.7% at 850 °C, which was even higher than for the derivatives with tricyanomethanide and dicyanamide anions at 850 °C. In contrast, [C_2_mim][DCA] left 33% of carbon residues at 450°C and 13.3% at 850 °C, while the carbohydrate-derived analog gave 20% and 6.3% respectively [32]. The highest difference was observed for the [Carb][TCM] derivative, which, at 450 °C, yielded 33% carbon residues, but, at 850 °C, decomposed completely into volatiles, while its imidazolium analog still gave 26% carbon residues.

### 2.3. Electrochemical Activity toward Oxygen Reduction Reaction (ORR)

**Figure 4:**
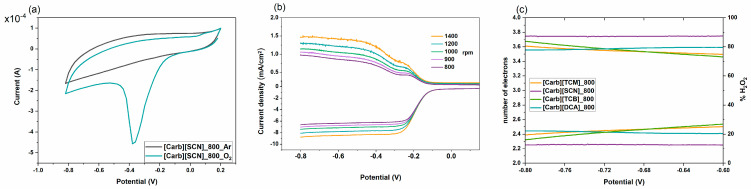
(**a**) CV curves recorded for [Carb][SCN]-derived carbon, deposited on a GC electrode in O_2_- and Ar-saturated 0.1 M KOH at a scan rate of 50 mV/s; (**b**) LSV recorded for [Carb][SCN]-derived carbon deposited on a GC electrode in O_2_-saturated 0.1 M KOH at a scan rate of 50 mV/s; (**c**) electron-transfer numbers and peroxide yields (H_2_O_2_%) calculated from RRDE measurements registered in O_2_-saturated 0.1 M KOH at a scan rate of 50 mV/s for carbohydrate-derived carbons synthesized at 800 °C.

The electrochemical activity of the prepared carbon materials in the ORR was determined by cyclic voltammetry (CV) and linear sweep voltammetry (LSV) in 0.1 M KOH solution (Figure 4). In the O_2_-saturated medium, the CV curves gave an irreversible cathodic peak at approximately −0.40 V (vs. Ag/AgCl), associated with the irreversible reduction of oxygen, as depicted for the representative [Carb][SCN]-derived carbon (Figure 4a), whereas, in the Ar-saturated solutions, for all the samples, quasi-rectangular voltammograms with no significant redox peak were recorded.

In the next series of experiments, the ORR electrocatalytic activity of the materials was investigated using a rotating ring-disc electrode (RRDE) at various rotating speeds (Figure 4b). The onset potential for the ORR varied between materials and generally increased with an increase in the carbonization temperature (Table 3). This enhanced activity can be ascribed to the increased amount of intrinsic carbon structural defects, which was confirmed by Raman spectroscopy (Figure 3c) [37]. Moreover, the highest onset potential, −0.12 V, was observed for the carbon material derived at 800 °C from the [Carb][SCN] precursor, also indicating the remarkable effect of N and S dual-doping [38]. Investigating more closely the mechanism of oxygen reduction by performing RRDE measurements, all the materials catalyzed the reaction, mainly through a ca. four-electron process (O_2_ to H_2_O), as shown by the number of electrons transferred (Figure 4c, Table 3), which was higher than 3.25 (for the material derived from [Carb][SCN], approaching even 3.74) over the range of −0.8 to −0.6 V. In fact, S and N dual-doped carbons gave higher electron transfer numbers than single N-doped carbons carbonized at the same temperature.

A significant influence on the activity of the materials derived from the carbohydrate-based ILs and salts was their high specific surface areas, achieved by using a hard template in the carbonization process. Even though the material without a template was carbonized at a higher temperature (900 °C), it gave both a lower onset potential for the ORR (−0.15 V) [5] than when carbonized in the presence of Ludox nanoparticles (−0.14 V) and a lower number of electrons transferred (3.15 vs. 3.56) under the same measurement conditions. The difference may be ascribed to the significant difference in the textural properties; the material carbonized in the presence of the silica template ([Carb][DCA]_800) gave an S_BET_ of ~700 m^2^·g^−1^, while when carbonized without a template gave only 146 m^2^·g^−1^.

## 3. Materials and Methods

^1^H NMR and ^13^C NMR spectra (Figure S1) were recorded with a 400 MHz Agilent spectrometer. Chemical shifts (ppm) are reported relative to tetramethylsilane as an internal standard. DMSO-*d*6 was used as solvent. High-resolution mass spectrometry analyses (Figure S2) were carried out on a Waters Xevo G2 Q-TOF mass spectrometer equipped with an ESI source operating in the positive and negative ion modes. The accurate mass and composition of the molecular ions were calculated using MassLynx software V4.1 SCN802 2011, Waters Inc., Etten-Leur, The Netherlands. Thermogravimetric analysis (TGA) was performed using a Mettler-Toledo TGA/DSC 3+ thermobalance. Samples were heated from 50 to 1000 °C, at a rate of 10 °C/min, in standard 70 μL Al_2_O_3_ crucibles under a dynamic flow of N_2_ (80 mL/min).

Raman spectra of the samples were recorded with a laser having an excitation wavelength of 514 nm and a 2400 lines/mm grating at room temperature using a Renishaw inVia Raman microscope. IR spectra (Figure S4) of the samples were collected in ATR (attenuated total reflectance) mode using a Perkin Elmer IR spectrometer. Nitrogen adsorption and desorption isotherms were obtained with a Micromeritics ASAP 2420M instrument at −196 °C. The specific surface area was calculated using the Brunauer–Emmett–Teller method. The size of the pores was obtained using the Barrett–Joyner–Halenda method with the Harkins and Jura equation and the Faas correction. Prior to the experiments, the samples were outgassed at 200 °C and 1.33 × 10^−3^ Pa for 5 h. Elemental analyses (C, N, S, and H content) were carried out using an Elementar VARIO EL III elemental analyzer, with the absolute deviation across the three measurements for each experiment being less than 0.4 for C, 0.07 for H, 0.02 for N, and 0.05 for S. The morphology of the samples was investigated by scanning electron microscopy (Phenom Pro Desktop SEM). Differential scanning calorimetry (DSC) was performed using a Perkin Elmer DSC 8000, calibrated using indium (Perkin Elmer, *T*_m_ = 156 °C, Δ*H*_f_ = 28.45 J·g^−1^). The obtained data were examined using Pyris software version 13, Waltham, MA, USA. Experiments were carried out under a nitrogen atmosphere in triplicate with a heating rate of 10 °C·min^−1^. The presented values were taken from the second cycle. The melting point (*T*_m_, °C) was considered to be the peak maximum, and the glass transition temperature (*T*_g_, °C) was taken from the heating cycle.

### 3.1. Materials

Methyl α-d-glucopyranoside (≥99%), tetrabromomethane (99%), triphenylphosphine (99%), anhydrous pyridine (99.8%), sodium dicyanamide (96%), trimethylamine 31–35 wt.% in ethanol solution, and Ludox^®^ HS-40 colloidal silica (40 wt.% suspension in H_2_O) were commercial materials purchased from Sigma-Aldrich. Potassium tricyanomethanide (98%) and potassium tetracyanoborate (97%) were acquired from Strem, Newburyport, MA, USA and SelectLab Chemicals, Münster, Germany respectively. Silver nitrate (99.9%) was received from POCH, Gliwice, Poland. *N*-(6-Deoxy-1-*O*-methoxy-α-d-glucopyranoside)-*N*,*N*,*N*-trimethylammonium bromide was synthesized according to a previously described procedure [5].

### 3.2. Carbonization of Ionic Precursors

Carbohydrate-derived precursors were carbonized with Ludox^®^ HS-40. The carbonization conditions were as follows: 0–750 °C (10°/min), 750–800 °C (1.7°/min), 800 °C (2 h), Ar 100 mL/min or 0–160 °C (4°/min), 160–600 °C (10°/min), 600 °C (2 h), Ar 100 mL/min. After carbonization, the silica was removed by washing with 4 M ammonium hydrogen difluoride (NH_4_HF_2_). For each 1 g of silica present in the obtained carbon materials, 45 mL of the NH_4_HF_2_ solution was used. The samples were denoted indicating the precursor used and carbonization temperature, e.g., [Carb][SCN]_800 for the material derived from [Carb][SCN] at 800 °C.

### 3.3. Synthesis of the Precursors

#### 3.3.1. *N*-(6-Deoxy-1-*O*-Methoxy-α-d-Glucopyranoside)-*N*,*N*,*N*-Trimethylammonium Thiocyanate [Carb][SCN]

*N*-(6-Deoxy-1-*O*-methoxy-α-d-glucopyranoside)-*N*,*N*,*N*-trimethylammonium bromide (17.59 g, 56 mmol) was dissolved in methanol (350 mL), and silver thiocyanate (9.49 g, 57 mmol) was added. The resulting suspension was covered from light and stirred for 24 h. The slurry was filtered, while the liquid phase was collected and the solvent was evaporated. A white solid was obtained (15.97 g, 98%, m.p. = 173 °C).

^1^H-NMR (400 MHz, DMSO) δ 5.45 (d, 1H, –OH; *J* = 6.0 Hz), 5.03 (d, 1H, –OH; *J* = 5.1 Hz), 4.92 (d, 1H, –OH; *J* = 6.4 Hz), 4.60 (d, 1H, H-1; *J* = 3.7 Hz), 3.89 (t, 1H, H-6b; *J* = 9.0 Hz), 3.63 (d, 1H, H-6a; *J* = 13.3 Hz), 3.41–3.53 (m, 2H, H-4, H-5) 3.39 (s, 3H, –OCH_3_), 3.19–3.24 (m, 1H, H-3), 3.13 (s, 9H, –N^+^(CH_3_)_3_), 2.88–2.95 (m, 1H, H-3). ^13^C-NMR (101 MHz, DMSO) δ 129.06 (–CN), 100.63 (C-1), 72.33, 71.54, 71.09 (C-2, C-3, C-4), 66.93, 66.79 (C-5, C-6), 56.40 (–OCH_3_), 53.32 (–N^+^(CH_3_)_3_). ESI-MS: [M^+^] calculated: 236.1498; found: 236.1504; [M^−^] calculated: 57.9751; found: 57.9757.

#### 3.3.2. *N*-(6-Deoxy-1-*O*-Methoxy-α-d-Glucopyranoside)-*N*,*N*,*N*-Trimethylammonium Dicyanamide [Carb][DCA]

*N*-(6-Deoxy-1-*O*-methoxy-α-d-glucopyranoside)-*N*,*N*,*N*-trimethylammonium bromide (14.35 g, 45 mmol) was dissolved in methanol (200 mL), and silver dicyanamide (7.89 g, 45 mmol) was added. The resulting suspension was covered from light and stirred for 24 h. The slurry was filtered, while the liquid phase was collected and the solvent was evaporated. A white solid was obtained (12.80 g, 94%, m.p. = 123 °C).

^1^H-NMR (400 MHz, DMSO) δ 5.45 (d, 1H, –OH; *J* = 6.0 Hz), 5.03 (d, 1H, –OH; *J* = 5.2 Hz), 4.92 (d, 1H, –OH; *J* = 6.4 Hz), 4.60 (d, 1H, H-1; *J* = 3.7 Hz), 3.89 (t, 1H, H-6b; *J* = 9.0 Hz), 3.63 (d, 1H, H-6a; *J* = 13.1 Hz), 3.41–3.52 (m, 2H, H-4, H-5), 3.39 (s, 3H, –OCH_3_), 3.19–3.24 (m, 1H, H-3), 3.12 (s, 9H, –N^+^(CH_3_)_3_), 2.90–2.94 (m, 1H, H-3). ^13^C-NMR (101 MHz, DMSO) δ 118.98 (–CN), 100.64 (C-1), 72.34, 71.55, 71.10 (C-2, C-3, C-4), 66.94, 66.80 (C-5, C-6), 56.40 (–OCH_3_), 53.32 (–N^+^(CH_3_)_3_). ESI-MS: [M^+^] calculated: 236.1498; found: 236.1498; [M^−^] calculated: 66.0092; found: 66.0104.

#### 3.3.3. *N*-(6-Deoxy-1-*O*-Methoxy-α-d-Glucopyranoside)-*N*,*N*,*N*-Trimethylammonium Tricyanomethanide [Carb][TCM]

*N*-(6-Deoxy-1-*O*-methoxy-α-d-glucopyranoside)-*N*,*N*,*N*-trimethylammonium bromide (9.58 g, 30 mmol) was dissolved in methanol (200 mL), and silver tricyanomethanide (6.00 g, 30 mmol) was added. The resulting suspension was covered from light and stirred for 24 h. The slurry was filtered, while the liquid phase was collected and the solvent was evaporated. A cream-colored viscous liquid was obtained (9.61 g, 97%).

^1^H-NMR (400 MHz, DMSO) δ 5.45 (d, 1H, –OH; *J* = 6.0 Hz), 5.04 (d, 1H, –OH; *J* = 5.2 Hz), 4.92 (d, 1H, –OH; *J* = 6.4 Hz), 4.60 (d, 1H, H-1; *J* = 3.7 Hz), 3.89 (t, 1H, H-6b; *J* = 9.0 Hz), 3.63 (d, 1H, H-6a; *J* = 13.2 Hz), 3.41–3.52 (m, 2H, H-4, H-5), 3.39 (s, 3H, –OCH_3_), 3.19–3.24 (m, 1H, H-3), 3.12 (s, 9H, –N^+^(CH_3_)_3_), 2.91–2.95 (m, 1H, H-2). ^13^C-NMR (101 MHz, DMSO) δ 120.38 (–CN), 100.66 (C-1), 72.36, 71.55, 71.10 (C-2, C-3, C-4), 66.94, 66.80 (C-5, C-6), 56.41 (–OCH_3_), 53.33 (–N^+^(CH_3_)_3_), 4.64 (C(CN)_3_^−^). ESI-MS: [M^+^] calculated: 236.1498; found: 236.1498; [M^−^] calculated: 90.0092; found: 90.0127.

#### 3.3.4. *N*-(6-Deoxy-1-*O-*Methoxy-α-d-Glucopyranoside)-*N*,*N*,*N*-Trimethylammonium Tetracyanoborate [Carb][TCB]

*N*-(6-Deoxy-1-*O*-methoxy-α-d-glucopyranoside)-*N*,*N*,*N*-trimethylammonium bromide (9.85 g, 31 mmol) was dissolved in methanol (200 mL), and silver tetracyanoborate (6.94 g, 31 mmol) was added. The resulting suspension was covered from light and stirred for 24 h. The slurry was filtered, while the liquid phase was collected and the solvent was evaporated. A yellow viscous liquid was obtained (10.55 g, 96%, m.p. = 76 °C).

^1^H-NMR (400 MHz, DMSO) δ 5.45 (d, 1H, –OH; *J* = 6.0 Hz), 5.04 (d, 1H, –OH; *J* = 5.1 Hz), 4.92 (d, 1H, –OH; *J* = 6.4 Hz), 4.60 (d, 1H, H-1; *J* = 3.7 Hz), 3.89 (t, 1H, H-6b; *J* = 9.0 Hz), 3.63 (d, 1H, H-6a; *J* = 13.2 Hz), 3.43–3.52 (m, 2H, H-4, H-5), 3.39 (s, 3H, –OCH_3_), 3.20–3.25 (m, 1H, H-3), 3.12 (s, 9H, –N^+^(CH_3_)_3_), 2.91–2.95 (m, 1H, H-2). ^13^C-NMR (101 MHz, DMSO) δ 122.68, 121.98, 121.27, 120.57 (–CN), 100.63 (C-1), 72.34, 71.53, 71.09 (C-2, C-3, C-4), 66.92, 66.79 (C-5, C-6), 56.38 (–OCH_3_), 53.31 (–N^+^(CH_3_)_3_). ESI-MS: [M^+^] calculated: 236.1498; found: 236.1498; [M^−^] calculated: 115.0216; found: 115.0238.

#### 3.3.5. Silver Thiocyanate

Potassium thiocyanate (17.54 g, 180 mmol) and silver nitrate (33.67 g, 198 mmol) were each dissolved in 140 mL of water. The silver nitrate solution was added dropwise to the potassium thiocyanate solution, with a brownish precipitate being immediately formed. The reaction was covered from the light and stirred for 24 h. The precipitated product was filtered off, washed with water, and dried (29.42 g, 98%).

#### 3.3.6. Silver Dicyanamide

Sodium dicyanamide (25.00 g, 281 mmol) and silver nitrate (47.70 g, 281 mmol) were each dissolved in 150 mL of water. The silver nitrate solution was added dropwise to the sodium dicyanamide solution, with a violet precipitate being immediately formed. The reaction was covered from light and stirred for 24 h. The precipitated product was filtered off, washed with water, and dried (25.30 g, 95%).

#### 3.3.7. Silver Tricyanomethanide

Potassium tricyanomethanide (4.02 g, 31 mmol) and silver nitrate (5.82 g, 34 mmol) were each dissolved in 40 mL of water. The silver nitrate solution was added dropwise to the potassium tricyanomethanide solution, with a cream precipitate being immediately formed. The reaction was covered from light and stirred for 24 h. The precipitated product was filtered off, washed with water, and dried (6.00 g, 97%).

#### 3.3.8. Silver Tetracyanoborate

Potassium tetracyanoborate (4.92 g, 32 mmol) and silver nitrate (5.97 g, 35 mmol) were each dissolved in 40 mL of water. The silver nitrate solution was added dropwise to the potassium tetracyanoborate solution, with a light-gray precipitate being immediately formed. The reaction was covered from light and stirred for 24 h. The precipitated product was filtered off, washed with water, and dried (6.94 g, 98%).

## 4. Conclusions

A group of new bio-derived ILs and salts, featuring cationic carbohydrates and cyano-based anions, were synthesized and investigated as doped carbon precursors. Combining α-d-glucopyranoside with thiocyanate [SCN], tricyanomethanide [TCM], and dicyanamide [DCA] anions had very little influence on the stability of the parent sugar, in contrast to the tetracyanoborate [TCB] anion, which provided the highest thermal stability (T_onset_ = 308 °C) for a carbohydrate-based IL reported so far. Possessing a high carbon content and cross-linkable anions, all the precursors yielded carbon residues upon carbonization, yet with mostly lower yields than their petroleum-derived analogs. The exception was the precursor possessing an inexpensive thiocyanate anion, which had a higher carbonization yield than reported for its imidazolium analog [C_2_mim][SCN], additionally facilitating the synthesis of S and N co-doped carbon. Dual-doping resulted in an enhanced catalytic activity of the carbohydrate IL-derived carbon toward ORR, demonstrated by a positively shifted onset potential and high electron transfer number, making it comparable to the commercial Pt/C catalyst.

## Figures and Tables

**Table 1:** Thermal properties of ionic products.

IL/Salt	*T*_g_ (°C ± 1 °C)	*T*_m_ (°C ± 1 °C)	*T*_onset_ (°C)	Notes
[Carb][SCN]	23	173	229	solid
[Carb][DCA]	10	123	232	solid
[Carb][TCB]	−5	76	308	solid, hygroscopic
[Carb][TCM]	−10	-	255	liquid, hygroscopic

**Table 2:** Chemical compositions obtained by elemental analysis and yield of the carbon materials.

Carbon Material	T (°C)	C Yield Exp. (wt.%)	C Yield Theor. (wt.%) ^a^	C Yield Theor. (wt.%) ^b^	N (%)	C (%)	H (%)	S (%)
[Carb][SCN]	500	25.9	4.1	26	8.5	65.7	3.4	4.7
[Carb][SCN]	600	25.7	4.1	24	7.9	69.1	2.0	3.0
[Carb][SCN]	800	19.4	4.1	19	5.9	71.6	1.7	2.6
[Carb][DCA]	600	32.8	7.9	11	11.1	73.6	2.6	-
[Carb][DCA]	800	17.9	7.9	14	8.7	79.4	2.0	-
[Carb][TCM]	600	29.9	14.7	22	11.1	74.7	2.5	-
[Carb][TCM]	800	24.8	14.7	22	7.1	76.7	1.6	-
[Carb][TCB]	600	21.4	13.7	15	8.2	78.3	2.5	-
[Carb][TCB]	800	28.1	13.7	15	6.1	69.1	1.7	-

^a^ Theoretical yield calculated on the basis of the overall carbon content in the precursor; ^b^ theoretical yield calculated on the basis of the overall carbon content in the anion.

**Table 3:** The electrochemical properties of the carbon materials.

Carbon Material	E_onset_ (V) *	No. of Electrons **	% H_2_O_2_ **
[Carb[SCN]_800	−0.12	3.74	12.8
[Carb[SCN]_600	−0.20	3.62	18.9
[Carb[SCN]_500	−0.29	3.42	28.9
[Carb][DCA]_800	−0.14	3.56	21.7
[Carb][DCA]_600	−0.29	3.25	37.2
[Carb][TCM]_800	−0.13	3.56	21.7
[Carb][TCM]_600	−0.25	3.65	17.6
[Carb][TCB]_800	−0.14	3.60	19.5
[Carb][TCB]_600	−0.22	3.42	28.9

* The onset potentials for ORR; ** electron-transfer numbers and peroxide yields (H_2_O_2_%) at E = −0.75 V calculated from RRDE measurement (1600 rpm, in O_2_-saturated 0.1 M KOH at a scan rate of 50 mV/s).

## Data Availability

The data presented in this study are available in article or Supplementary Materials.

## Supplementary Materials

^1^H and ^13^C NMR, MS, IR spectra and DSC thermograms are available online at https://www.mdpi.com/article/10.3390/ijms221910426/s1.
