# Supplementary material for: Bioderived Ionic Liquids and Salts with Various Cyano Anions as Precursors for Doped Carbon Materials
Source: Int J Mol Sci. 2021 Sep 27;22(19):10426. doi: 10.3390/ijms221910426 (PMC8508855; doi:10.3390/ijms221910426)

Supporting Information

Bio-derived ionic liquids and salts with various cyano anions as precursors for doped carbon materials

Alina Bryczek-Szafran [1,*], Bartłomiej Gaida [1], Agata Blacha-Grzechnik[1], Karolina Matuszek[2], and Anna Chrobok[1]

1 Faculty of Chemistry, Silesian University of Technology, Krzywoustego 4, Gliwice, 44-100, Poland

2 School of Chemistry, Monash University, Clayton 3800, VIC, Australia

* Correspondence: alina.brzeczek-szafran@polsl.pl

Figure S1. ^{1}H and ^{13}C NMR spectra of the investigated carbohydrate-derived ionic liquids and salts.

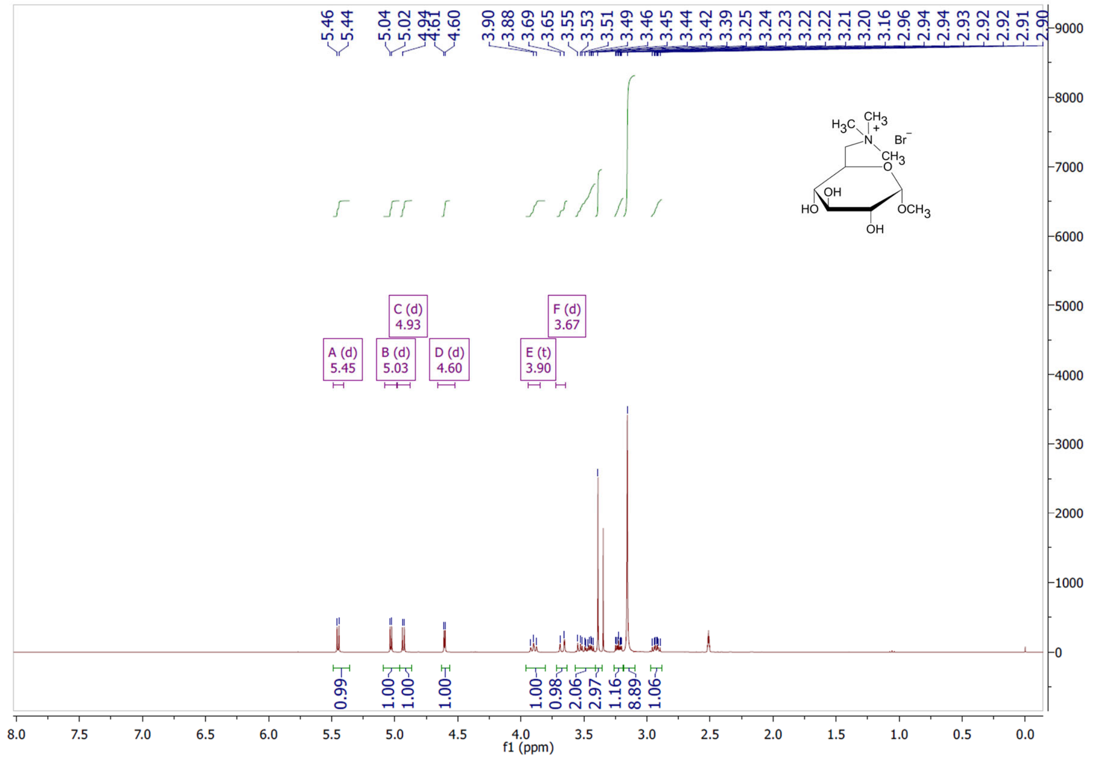

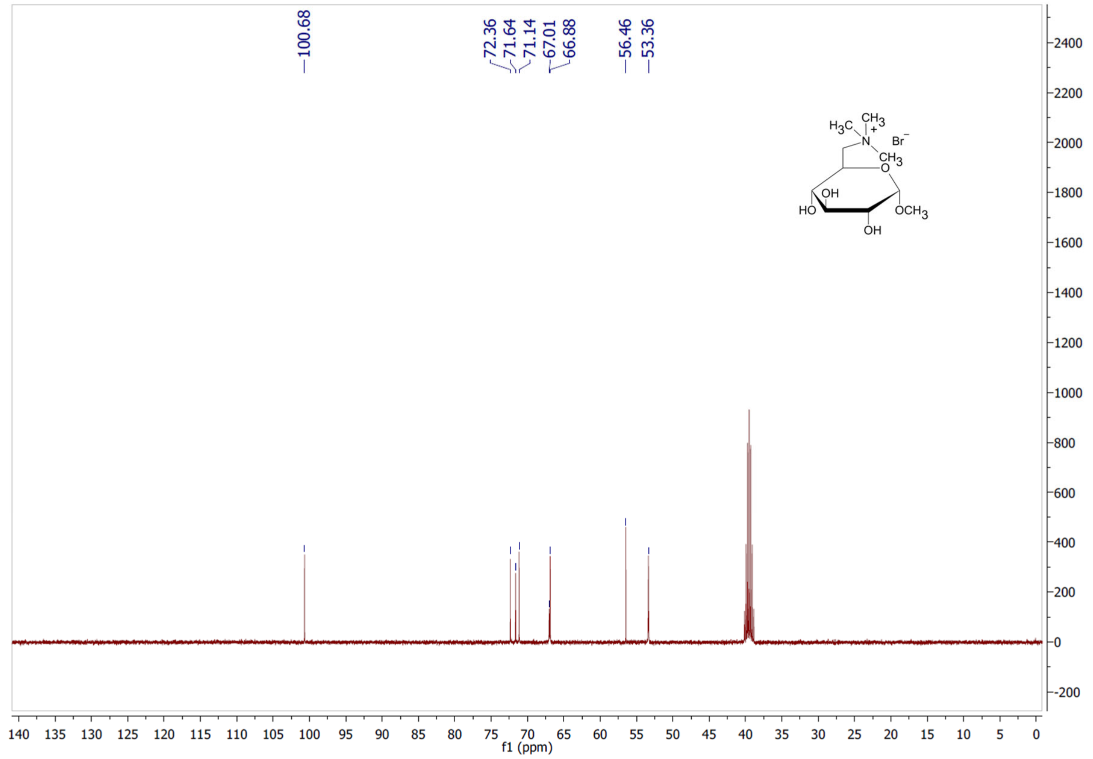

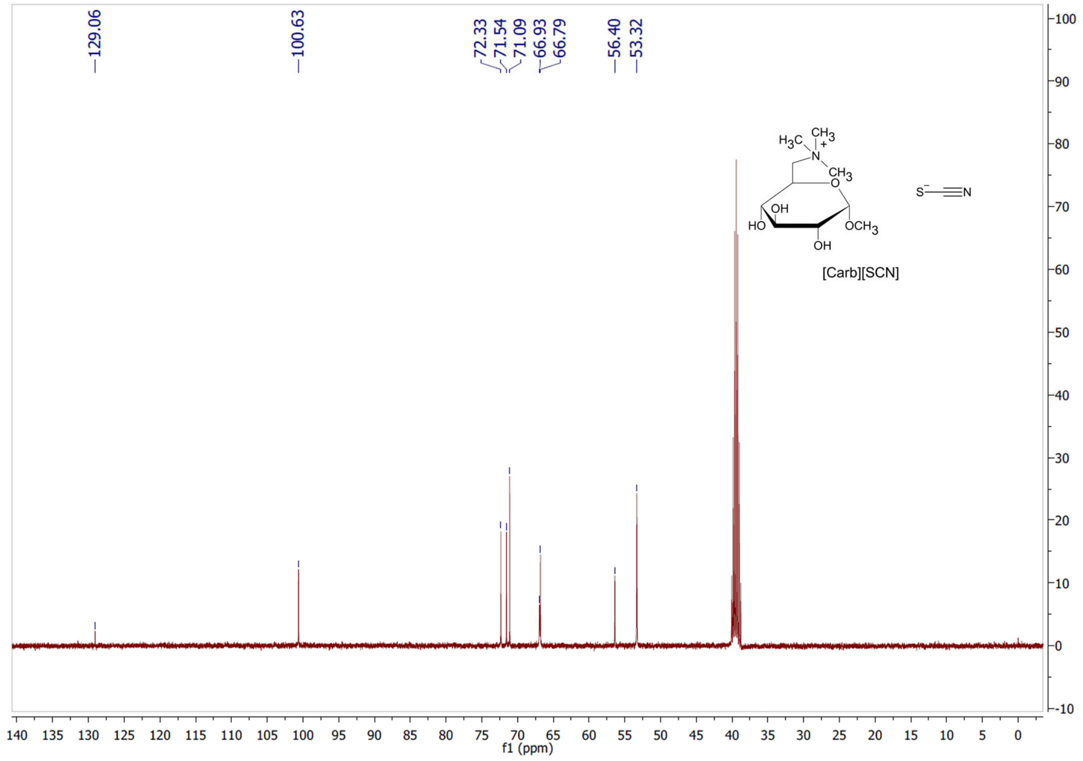
129.06
100.63
72.33
71.54
71.09
66.93
66.79
56.40
53.32
[Carb][SCN]
f1 (ppm)

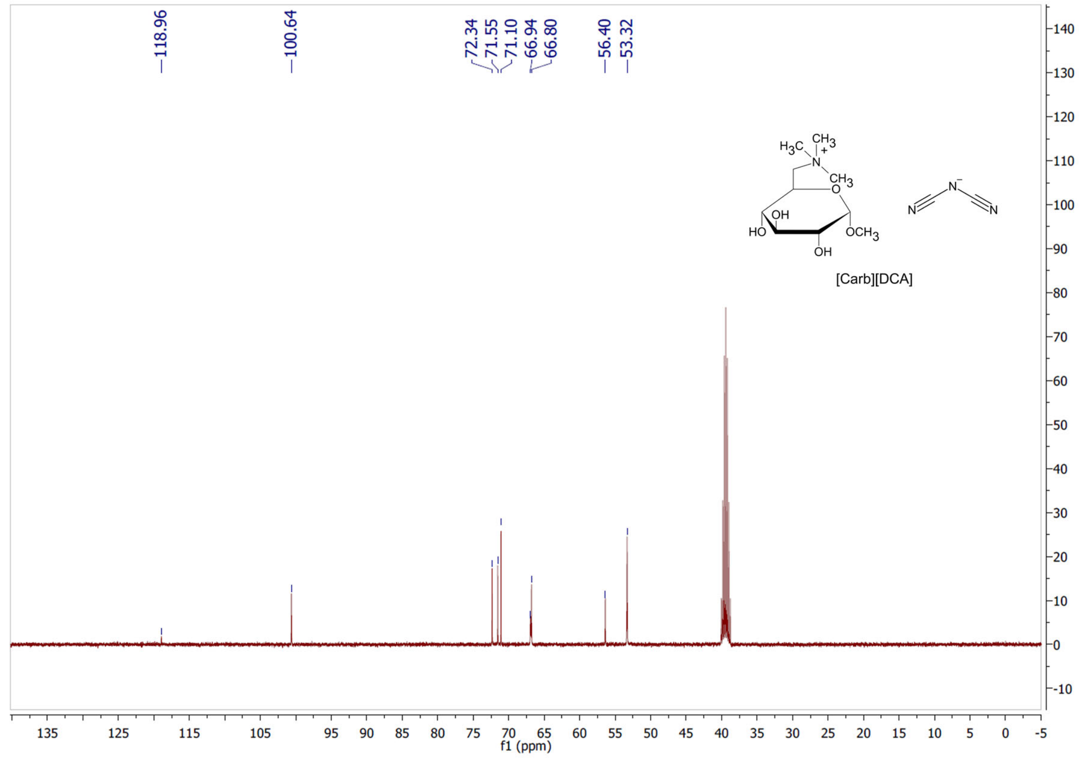
118.96
100.64
72.34
71.55
71.10
66.94
66.80
56.40
53.32
[Carb][DCA]
f1 (ppm)

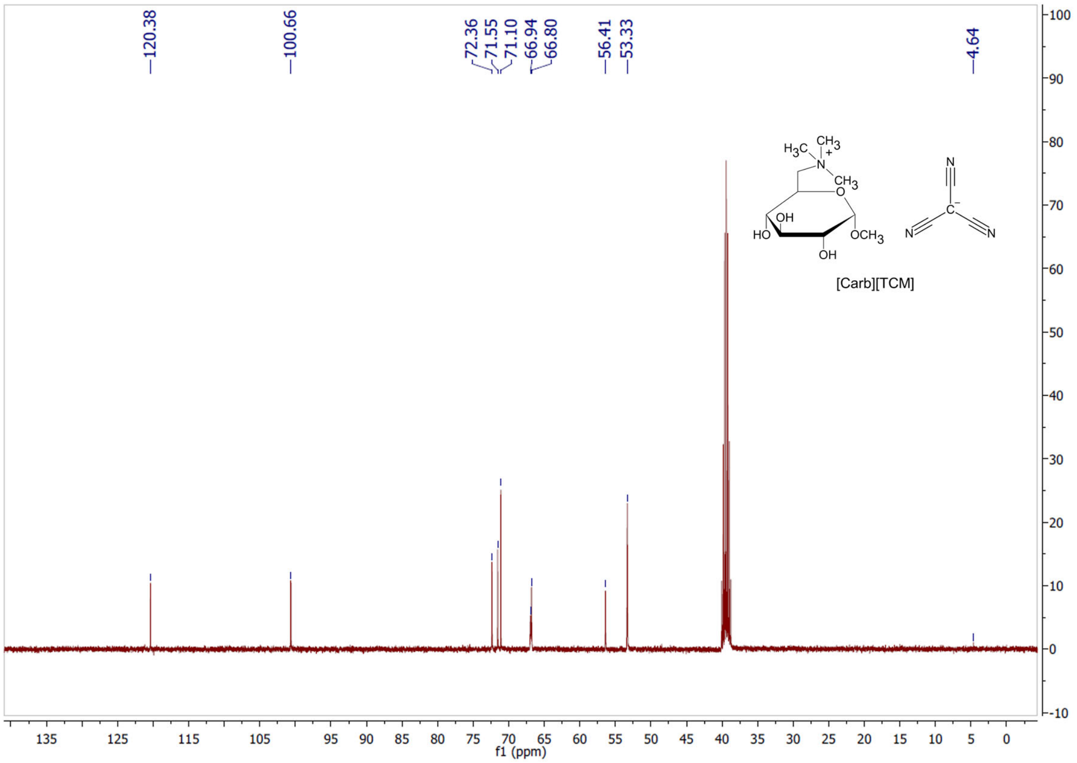
120.38
100.66
72.36
71.55
71.10
66.94
66.80
56.41
53.33
4.64
[Carb][TCM]
f1 (ppm)

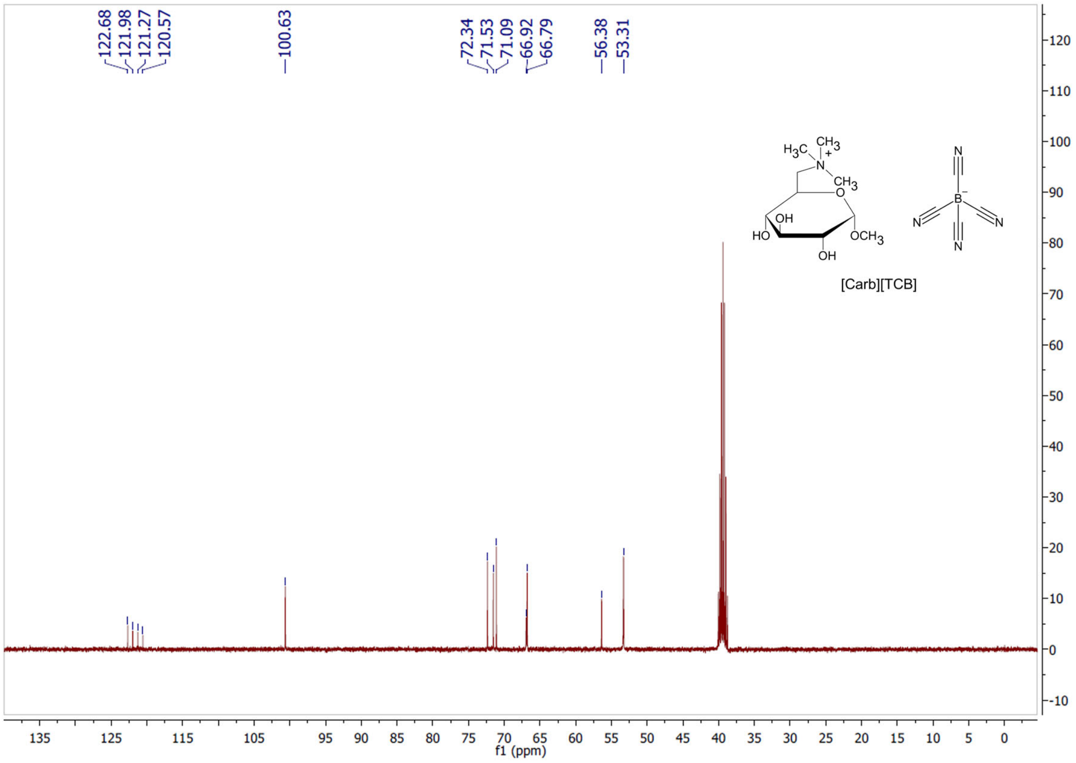
122.68
121.98
121.27
120.57
100.63
72.34
71.53
71.09
66.92
66.79
56.38
53.31
[Carb][TCB]
f1 (ppm)

Figure S2. MS spectra of the investigated carbohydrate-derived ionic liquids and salts.

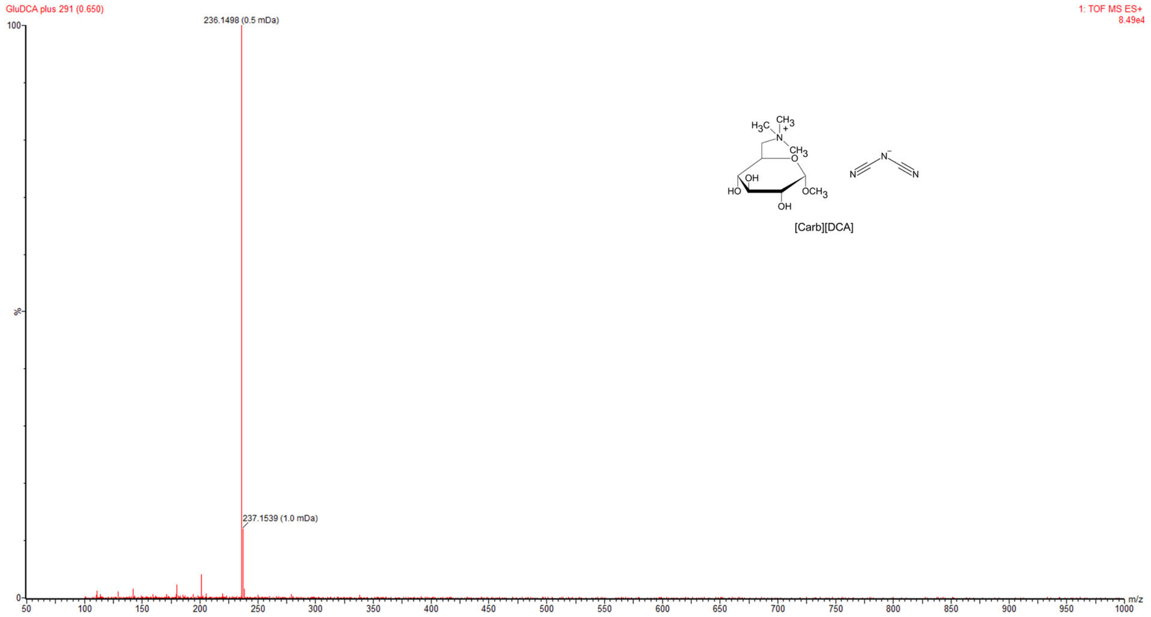

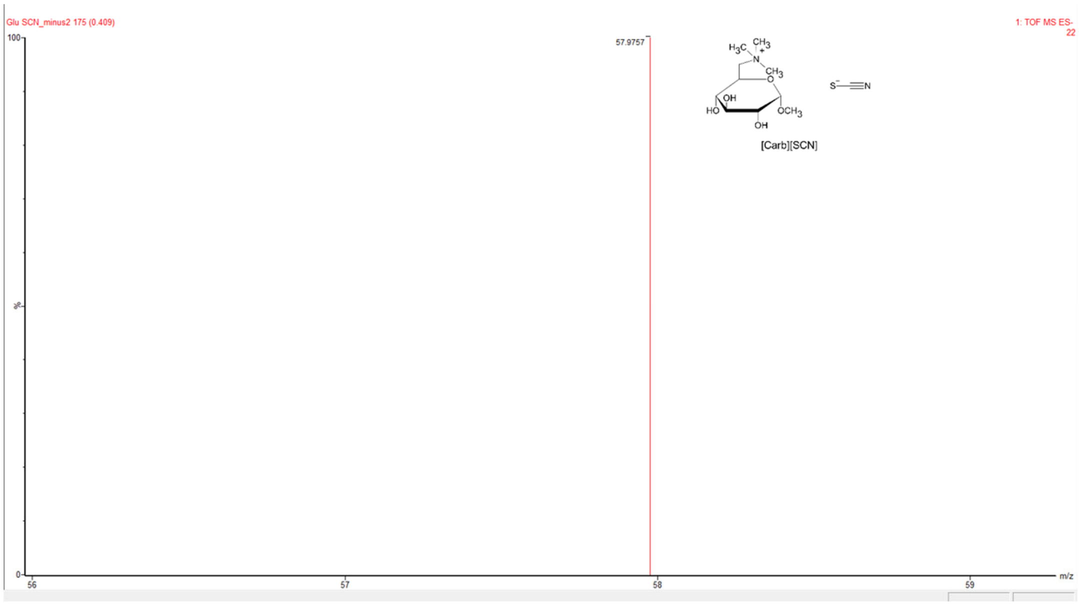

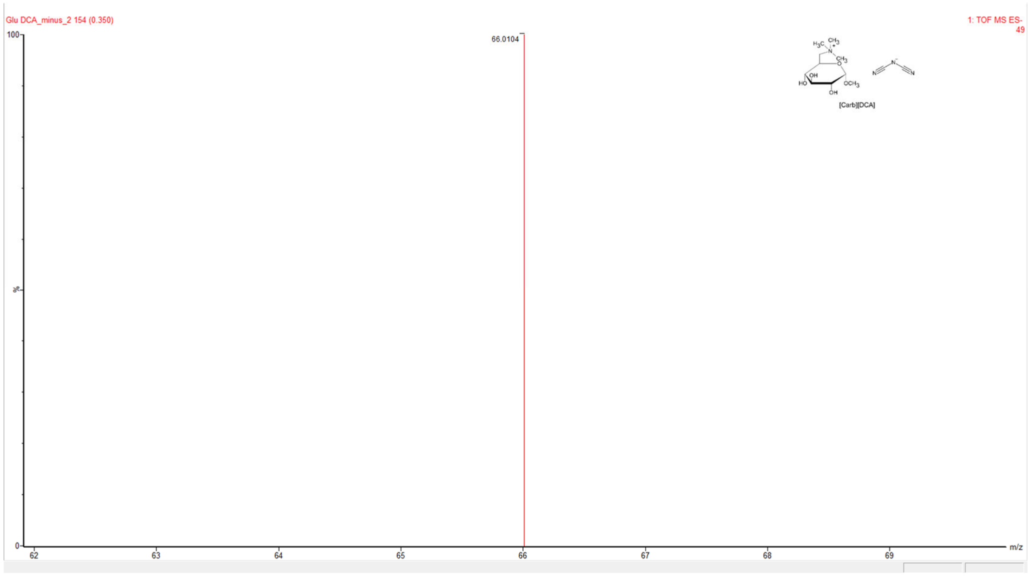
Glu DCA_minus_2 154 (0.350)
1: TOF MS ES-
49
100
66.0104
[Carb][DCA]
%
0
62
63
64
65
66
67
68
69
m/z

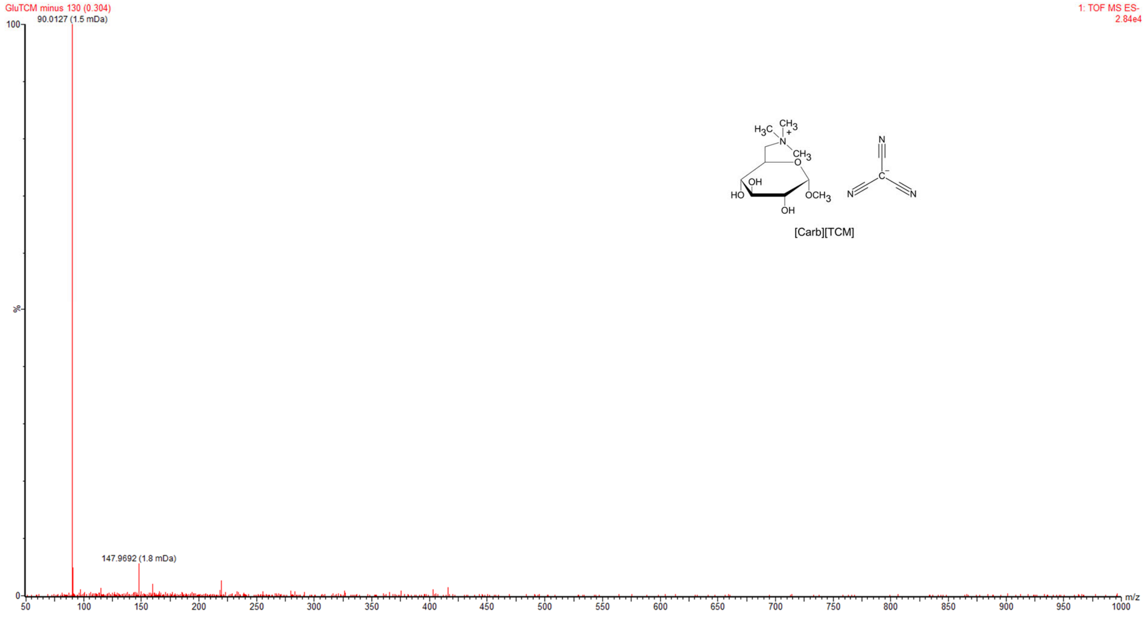
GluTCM minus 130 (0.304)
1: TOF MS ES-
2.84e4
100
90.0127 (1.5 mDa)
[Carb][TCM]
%
147.9692 (1.8 mDa)
0
50
100
150
200
250
300
350
400
450
500
550
600
650
700
750
800
850
900
950
1000
m/z

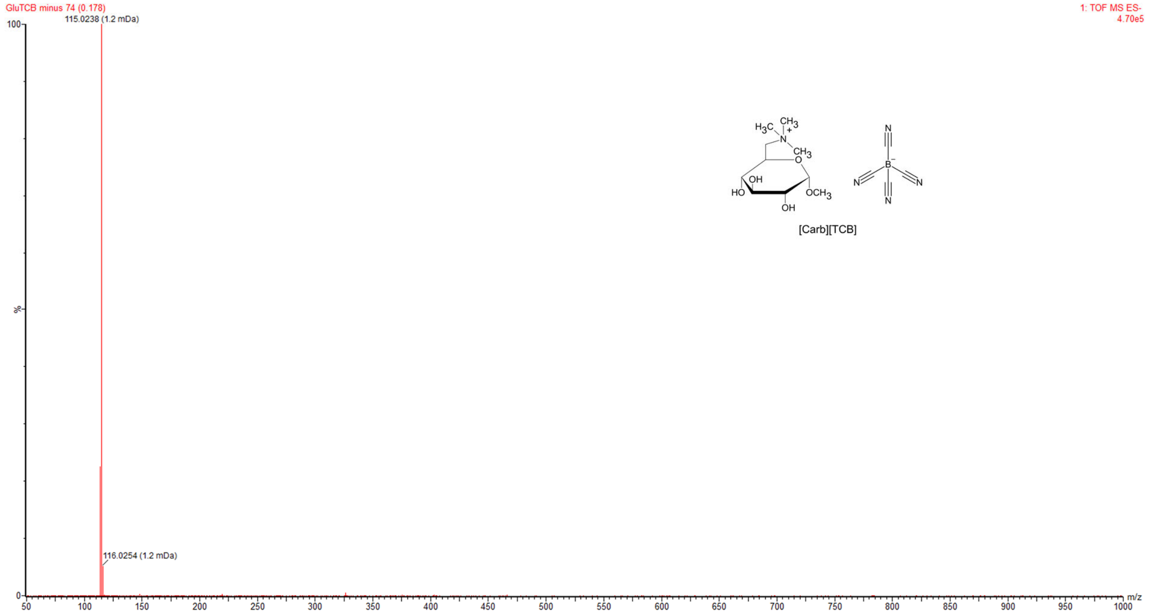

Figure S3. Thermal properties of carbohydrate-derived ionic liquids and salts: a) [Carb][DCA]; b)[Carb][SCN], c) [Carb][TCB], d)[Carb][TCM]

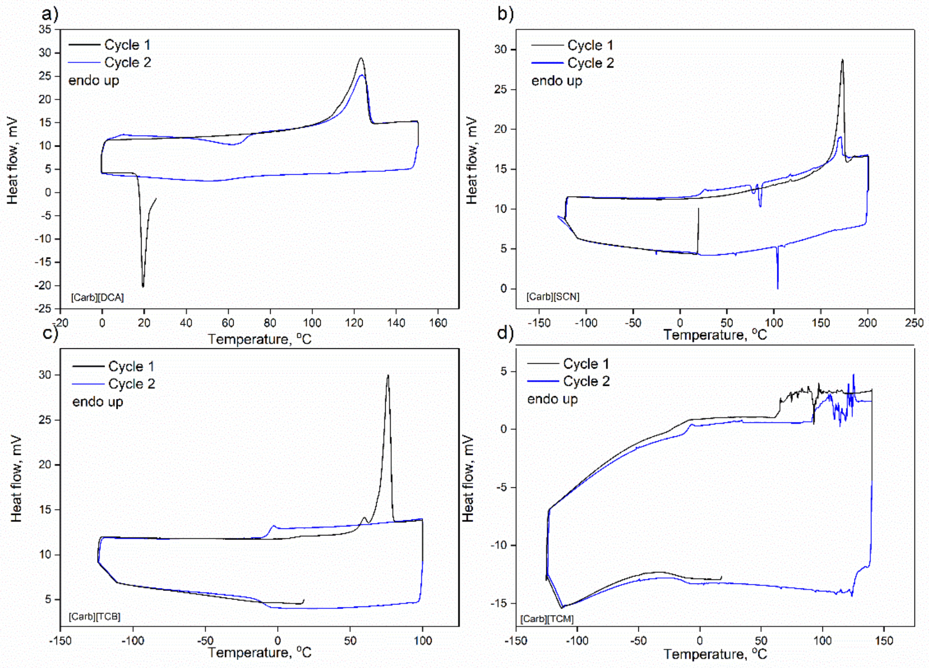

Figure S4. IR spectra of [Carb][SCN]_500 and [Carb][SCN]_600.

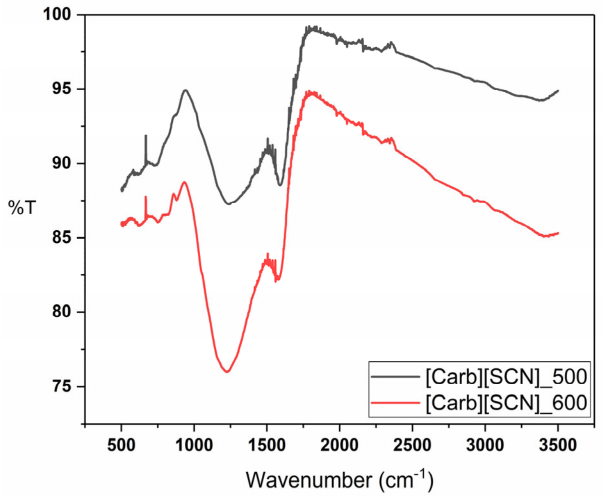

Supplement: Supplementary file 1 [file ijms-22-10426-s001.zip › ijms-1387758-supplementary.pdf]
